# Marine Toxins and Nociception: Potential Therapeutic Use in the Treatment of Visceral Pain Associated with Gastrointestinal Disorders

**DOI:** 10.3390/toxins11080449

**Published:** 2019-07-31

**Authors:** Andreina Baj, Michela Bistoletti, Annalisa Bosi, Elisabetta Moro, Cristina Giaroni, Francesca Crema

**Affiliations:** 1 Department of Medicine and Surgery, University of Insubria, 21100 Varese, Italy; 2 Department of Internal Medicine and Therapeutics, University of Pavia, 27100 Pavia, Italy

**Keywords:** visceral pain, irritable bowel syndrome, inflammatory bowel disease, marine toxins, VGSCs, VGCCs, ASICs, TRPs, GABA_B_

## Abstract

Visceral pain, of which the pathogenic basis is currently largely unknown, is a hallmark symptom of both functional disorders, such as irritable bowel syndrome, and inflammatory bowel disease. Intrinsic sensory neurons in the enteric nervous system and afferent sensory neurons of the dorsal root ganglia, connecting with the central nervous system, represent the primary neuronal pathways transducing gut visceral pain. Current pharmacological therapies have several limitations, owing to their partial efficacy and the generation of severe adverse effects. Numerous cellular targets of visceral nociception have been recognized, including, among others, channels (i.e., voltage-gated sodium channels, VGSCs, voltage-gated calcium channels, VGCCs, Transient Receptor Potential, TRP, and Acid-sensing ion channels, ASICs) and neurotransmitter pathways (i.e., GABAergic pathways), which represent attractive targets for the discovery of novel drugs. Natural biologically active compounds, such as marine toxins, able to bind with high affinity and selectivity to different visceral pain molecular mediators, may represent a useful tool (1) to improve our knowledge of the physiological and pathological relevance of each nociceptive target, and (2) to discover therapeutically valuable molecules. In this review we report the most recent literature describing the effects of marine toxin on gastrointestinal visceral pain pathways and the possible clinical implications in the treatment of chronic pain associated with gut diseases.

## 1. Introduction

Visceral pain commonly refers to pain arising from internal organs within the thorax and abdomen, which, in its acute form, has usually an identifiable cause, such as infection or tissue injury, and is pharmacologically curable [1]. Conversely, as occurs for other types of chronic pain, chronic visceral pain is long lasting and drug therapies may be unsuccessful. Chronic visceral pain represents one of the main symptoms of functional gastrointestinal disorders, which lack a recognizable pathological cause, comprising irritable bowel syndrome (IBS) [2]. In addition, inflammatory bowel disease (IBD) may be also associated with long-lasting pain perception despite resolution of the underlying inflammatory state [3]. Visceral sensitivity is commonly not restricted to the affected organ, but is associated with both an expansion of the body area from which visceral pain is typically referred and an increased tenderness to cutaneous/subcutaneous examination of the referral area [2,4]. The scarce density of visceral sensory innervation and the organization of visceral afferent inputs to the central nervous system (CNS) largely explain why visceral pain is not commonly felt at the source, is diffused in character, and is generally associated with greater autonomic and emotional responses than is pain arising from superficial structures [4]. Indeed, chronic abdominal pain is difficult to localize unless adjacent structures, such as the parietal peritoneum or pleura, are involved. The mechanisms underpinning development of chronic visceral pain largely remain to be uncovered, compared with those underlying somatic pain. Increasing evidence suggests that chronic visceral pain represents a multifactorial process involving dysfunction of the “gut–brain axis” [5]. The latter is represented as a bidirectional communication system connecting the gut with the brain, which is fundamental not only for understanding the mechanisms underlying development of visceral pain, but also to explain the putative influence of psychosocial factors on gastrointestinal sensitivity. Neuronal circuitries within the CNS, the autonomic nervous system (ANS) and the enteric nervous system (ENS), the neuroendocrine hypothalamo–pituitary–adrenal axis, immune signals, and signals deriving from the gut microbiota represent the key players of this communication axis [5,6]. Research in this field opens an exciting potential scenario in which new molecules displaying high efficacy in the treatment of chronic visceral pain associated with gut disorders may be discovered. Indeed, treatment of chronic abdominal visceral pain is a fundamental issue, owing to the increasing incidence of functional and inflammatory gut disorders worldwide [2,7]. Available drugs are analgesics, also used for somatic pain treatment, such as opioids and nonsteroidal anti-inflammatory drugs (NSAIDs), benzodiazepines, antidepressants, or antispasmodics [2,3]. However, these drugs exert several effects on the body other than nociception, and may worsen symptoms associated with the disease. One of the possible approaches to overcome adverse actions of these conventional therapies is to peripherally block pain generation by affecting the function of target receptors and ion channels located on peripheral terminals and along axons of primary afferent neurons [8]. These mainly comprise ion channels, such as transient receptor potential (TRP), voltage-gated sodium channels (VGSC), voltage-gated calcium channels (VGCC), and acid-sensing ion channels (ASICs). Natural toxins, including marine toxins, owing to their ability to both bind and/or indirectly control the function of these nociceptive targets may represent important tools for the development of new analgesics for the treatment of visceral pain acting on ion channels and receptors [9,10,11]. In the first part of this review, we give an overview of the physiological and pathological basis of visceral pain perception, in particular regarding IBS and IBD pain symptoms. We then focus on the current knowledge concerning marine toxins as possible modulators of visceral pain perception.

## 2. Neurophysiology of Visceral Pain

### 2.1. CNS Modulation of Visceral Pain 

**Figure 1:**
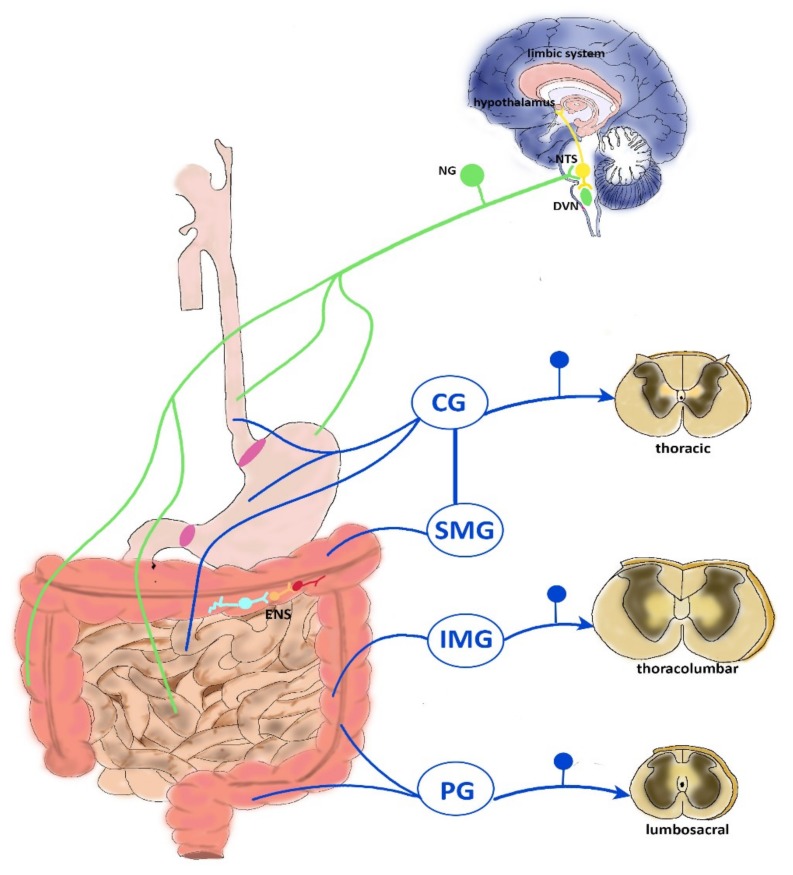
Schematic representation of visceral afferent pathways from the gastrointestinal tract. Vagal pathways (green) have their cell bodies in the nodose ganglion (NG) and nerve endings in the nucleus of the solitary tract (NTS) in the brain stem, and transduce signals prevalently originating from the upper gut, small intestine, and ascending colon. Efferent signals from vagal pathways (not shown) originate in the dorsal vagal nucleus (DVN). Splanchnic spinal thoracolumbar projections (blue) and spinal lumbosacral projections constituting the pelvic innervation (blue) have their soma in the dorsal root ganglia (DRG) and pass through prevertebral ganglia (celiac ganglion (CG); superior mesenteric ganglion (SMG), inferior mesenteric ganglion (IMG), and pelvic ganglion (PG)). Intrinsic primary neurons (light blue) are present within the enteric nervous system (ENS) and initiate appropriate motor, secretory, and vasomotor local reflex responses involving intrinsic interneurons (orange) and motor neurons (red). Visceral pain stimuli are conveyed to higher centers including the hypothalamus and limbic system, playing an important role in integrating visceral sensory and emotional information and higher order control of autonomic visceromotor responses.

Gastrointestinal sensory information is transmitted to the brain mainly via vagal, splanchnic spinal thoracolumbar projections, and spinal lumbosacral projections, constituting the pelvic innervation [12,13], as depicted in Figure 1. Extrinsic primary afferent pathways involved in bowel sensations include pathways ascending in both the anterolateral quadrant of the white commissure and in the dorsal column of the dorsal horn. In the spinal cord, extrinsic fibers impinge predominantly on the superficial part of laminae I and II, but also reach deeper layers, such as the laminae V–VII and X of the gray matter [13]. Vagal afferents project centrally to the nucleus of the solitary tract (NTS) in the brain stem, which, in turn, projects to the thalamus and, directly, the hypothalamus, locus coeruleus, amygdala, and periaqueductal grey [14,15]. First-order spinal afferent nerves synapse in the dorsal horn of the spinal cord with second-order neurons projecting to the brain through the spinoreticular, spinomesencephalic, spinohypothalamic, and spinothalamic pathways ascending in the anterolateral quadrant. The first three of these ascending tracts project to the brainstem in the dorsal reticular nucleus, to the periaqueductal gray and other midbrain regions, and to the hypothalamus, respectively, and largely mediate unconscious and/or automatic responses to visceral sensory input such as arousal, orientation, autonomic responses, prototype behavioral, and emotional responses, including the emotional component of pain [5,15,16]. The spinothalamic tract terminates in the medial and posterior thalamus, from which the thalamocortical fibers project to the primary somatosensory cortex. The thalamus is the relay station where multiple somatic and visceral inputs, including nociception, converge before reaching the cortex [16]. The major cortical regions involved in the elaboration of visceral pain stimuli from the thalamus are the somatosensory cortices (SI/SII) (lateral pain system), the anterior cingulate cortex (ACC) (medial pain system), and the insula. In these cortical regions, conscious and more complex pain processing takes place: SI/SII cortices provide information on the intensity and localization of the stimulus, the ACC and the orbital prefrontal cortex mainly process the affective–motivational pain dimension, while the insula is the interoceptive cortex, where all information about the internal state of the organism is processed, playing an important role in integrating visceral sensory and emotional information and higher order control of autonomic visceromotor responses [15,16]. Higher supraspinal structures, including the nucleus raphe magnus, the periventricular gray of the hypothalamus, and the midbrain periaqueductal gray exert a modulatory role on spinal pathways via descending pathways. At the cortical level, the ACC is the most important source of descending modulatory pathways, projecting to the amygdala and the periaqueductal gray, which is considered the key pain modulatory region. Descending pathways fine-tune the pain sensory input in both an inhibitory and an excitatory way [5,17].

### 2.2. Vagal Innervation

The vagus nerve innervates the entire gut, except the transverse and distal colon. The vagal cell bodies reside in the nodose vagal ganglion (NG), and nerve endings in the CNS terminate in the NTS in the dorsal medulla. Vagal afferents mainly regulate feeding behavior by modulating upper gut reflexes e.g., gastric accommodation, gastric emptying, gastric/pancreatic secretion, emesis. In the mouse gut, three types of vagal fibers have been characterized: mechanoreceptors, tension receptors, and mucosal varicose nerve endings [12]. Vagal mechanical afferents terminate within the muscle layers and comprise intramuscular arrays and intraganglionic laminar endings. Intramuscular arrays innervate the longitudinal or circular muscle layers and respond to muscle stretch. Intraganglionic laminar endings impinge on the connective tissue surrounding myenteric ganglia and are sensitive to distension and muscle contraction. The tension-sensitive afferents, which are principally sensitive to physiological distension, are activated by normal peristaltic activity [18,19]. Vagal mucosal afferents have been detected in the stomach mucosa and in the villus and crypts of the small intestine mucosa, and serve to detect chemical stimuli, such as gastric acid, to regulate satiety, to allow food particle passage into the duodenum, and to sense local hormones secreted by the mucosa to start vagal reflexes [12]. Overall, vagal afferents control the physiological perception of mechanical stimuli, whereas pain evoked by distension of the upper gastrointestinal tract is probably mediated via splanchnic afferents. However, vagal afferents have been shown to participate in pain reactions evoked by chemical stimuli, such as gastric acid challenge [20]. In addition, activation of lower gut vagal afferents has been suggested to modulate visceral pain spinal transmission, since, after subdiaphragmatic vagotomy in rats, pain responses to colorectal distension increased [17]. Efferent vagal pathways originate in the dorsal motor nucleus of the vagus (DVN) and the nucleus ambiguous of the brain stem, and underlie control of motor and secretory gut functions [5].

### 2.3. Thoracolumbar Innervation

Splanchnic spinal afferent neurons, mainly consisting of unmyelinated C fibers, form the major pathway for visceral perception and have their cell bodies in the dorsal root ganglia (DRG). The thoracolumbar spinal cord (T1–L3) nerves innervate the entire gastrointestinal tract and are the functional counterpart of vagal and pelvic nerves. Data from animal studies indicate that splanchnic afferents represent the most important pathways transducing nociception from the gut [21]. In the upper gastrointestinal tract, splanchnic afferents mediate gastric mechano-nociception, but not gastric chemo-nociception, which is signaled by vagal sensory neurons. In the lower portion of the gut, splanchnic afferents are responsible for abdominal discomfort perception. In mice, splanchnic afferents of the colon prevalently innervate in the serosa (36%) and mesenteric (50%) membranes associated with mesenteric blood vessels. Similarly, in the mucosa, spinal varicose afferent axons often branch near submucosal blood vessels [17]. A vast majority of spinal thoracolumbar pain afferent neurons express neuropeptides such as calcitonin gene related peptide (CGRP), tachykinins, and the TRP for vanilloid (TRPV1) [22,23]. Splanchnic visceral afferent nerves project to the spinal cord via prevertebral ganglia (celiac, superior, and inferior mesenteric ganglion), where they may synapse with efferent sympathetic neurons. Sympathetic efferents start in the intermediolateral columns of the spinal cord and terminate in pre-vertebral ganglia, from which sympathetic nerves depart to innervate four primary targets: myenteric ganglia, submucosal ganglia, blood vessels, and sphincter muscle. As a rule, the sympathetic extrinsic innervation exerts an inhibitory influence on enteric neurons, slowing gastrointestinal transit [12].

### 2.4. Pelvic Innervation

Pelvic nerves and the sacral plexuses mainly innervate pelvic structures, such as the colorectum, the bladder, and the reproductive organs. Pelvic afferents have their cell bodies in the lumbosacral dorsal root ganglia and transfer visceral information into the L6–S2 lumbosacral segments of the spinal cord [24]. Pelvic nerves contain serosal, mucosal, and muscular (e.g., intraganglionic laminar ending and intramuscular arrays) afferents from the gut and are sensitive to circular stretch, which represents the primary stimulus generated by low-intensity colorectal distension or stool passage [25]. Similarly to the vagal afferent system, pelvic afferents transmit physiological sensation (e.g., urgency, desire to defecate, etc.) [12]. However, unlike vagal afferent fibers, pelvic afferents include pain fibers, since colorectal distension in rats induced a visceromotor reflex contraction of abdominal muscle by stimulating pain pathways of pelvic origin [21,26]. Hence, pelvic nerves are involved in normal physiological functions and acute pain, rather than in inflammatory pain, which is more specifically mediated by thoracolumbar spinal afferents. However, in rats, pelvic fiber sensitization was demonstrated after application of a chemical irritant to the colon, posing a role in nociception of pelvic nerves during inflammation [17]. Pre-enteric efferent pelvic pathways have their cell bodies in the sacral spinal cord and in pelvic ganglia, and provide motor control for the defecation reflex [27].

### 2.5. ENS Reflexes

The ENS is a complex neuronal network extending from the esophagus to the anal sphincter that controls motility, gastric secretion, transport of fluids across the epithelium, blood flow, and nutrient absorption [12]. Modulation of these functions originates from the integration of reflexes within the ENS with those mediated by sympathetic ganglia and reflexes conveyed from the gut to the CNS, via vagal, splanchnic, and pelvic nerves [12,17]. However, the ENS is almost autonomously able to influence small intestine and colonic responses, such as propulsion of intraluminal contents (peristaltic reflex) or formation and propagation of migrating myoelectric complexes during prolonged interdigestive fasting periods, with respect to the CNS and the ANS [28]. The ENS is composed of a vast number of neurons, 200–600 million neurons in humans. At least 20 distinct neuronal cell types have been classified in the ENS, according to their morphology, neurochemical coding, electrophysiological and functional properties, and projections (intrinsic primary afferent neurons IPANs, interneurons, and excitatory and inhibitory motor neurons). Enteric neurons concur to the formation of three major plexuses: the subserous, the myenteric (located between the circular and longitudinal smooth muscle layers), and the submucosal (located in the homonymous layer) plexuses [12]. IPANs have been morphologically identified as multi-axonal sensory neurons, with axons extending and branching in the lamina propria of the mucosa and axons entering the myenteric and submucosal ganglia, where they impinge on other IPANs, interneurons, and motor neurons. In the submucosal and myenteric plexus of the small and large intestine, IPANs represent 10–30% of neurons, and display peculiar electrophysiological properties characterized by broad sodium and calcium currents followed by early and late afterdepolarization potentials, which are separated by a depolarizing current [29]. IPANs respond to (1) chemical stimuli (i.e., entero-endocrine products, such as 5-hydroxytryptamine in the lumen), (2) mechanical distortion of the mucosa, and (3) mechanical force exerted on their processes either within the myenteric plexus or after stretching or contraction of the musculature, in order to initiate appropriate motor, secretory, and vasomotor reflex responses [12]. The chemical coding of IPANs is highly conserved in different animal species and is represented by cholinergic neurons, serotoninergic neurons, peptidergic neurons containing tachykinins and CGRP, and glutamatergic neurons [29,30]. Excitatory and inhibitory motor neurons receive fast excitatory synaptic potentials and innervate the gut longitudinal and circular smooth muscle layers and muscularis mucosae [31]. The primary neurotransmitters for excitatory motor neurons are acetylcholine (ACh) and tachykinins, while inhibitory motor neurons contain several neurotransmitters, including nitric oxide (NO), vasoactive intestinal peptide (VIP), and purinergic transmitters, although NO is considered the primary transmitter [12,32,33,34]. Local intestinal reflexes are coordinated by several types of ascending and descending interneurons, characterized by different chemical coding, depending on their projection and function [12]. Enteric neurons may establish a distinguishing cross-talk with different cell types of the enteric microenvironment, including enteric glial cells, smooth muscle cells, and the interstitial cells of Cajal, which are considered intestinal pacemaker cells, immunocytes of the gut-associated lymphoid tissue (which represents the most important immune cell reservoir of the human body), enterochromaffin cells, and microbes of the commensal flora [35,36,37,38]. Changes in the enteric microenvironment may influence IPAN excitability, which stringently controls the threshold and intensity of enteric reflexes. Alterations of IPAN properties have been related, for example, to development of hypersensitivity and dysmotility in functional gut disorders, promoting these neurons as potential targets for therapeutic compounds [39].

## 3. Chronic Visceral Pain: The Paradigms of IBS and IBD 

Chronic visceral pain is a frequent and incapacitating disorder with high prevalence rates worldwide and critical economic impact [1]. Functional gut disorders, such as IBS, are the most prevalent forms of visceral pain. IBS is a complex disease characterized by alterations of bowel habits, abdominal pain and distension, and is often associated with psychological disorders [2]. Indeed, the main cause of medical consultation in IBS patients is a severe and recurrent pain sensation having a deep impact on patient’s quality of life, depending on three main aspects: alterations of gut motility, development of visceral hypersensitivity, and psychological aspects [2]. In non-pathological conditions, changes in gut function, such as gastrointestinal distensions and contractions, are not associated with pain. However, in IBS patients, enhanced perception of mechanical triggers applied to the bowel, resulting in pain and discomfort, frequently develops, representing a form of visceral hypersensitivity [40]. Epidemiological data demonstrate that visceral hypersensitivity and related pain in IBS patients has a prevalence ranging from 33% to 90%, and correlates with higher severity of complications [41]. A low-grade inflammatory state, associated with the release of several inflammation mediators, such as neuropeptides, cytokines, and prostanoids, has been suggested as a possible mechanism underlying stimulation and sensitization of sensory afferent nerve endings, triggering visceral hypersensitivity [42]. This hypothesis is in line with the observation that in IBS patients, the incidence and severity of enhanced visceral perception is related to the amount of activated mast cells in proximity to enteric nerves [43]. Accordingly, pro-inflammatory cytokines may influence enteric sensory innervations, thus altering visceral sensory perception and pain threshold with consequent pain generation [44]. Interestingly, pain is also one of the presenting symptoms in up to 70% of patients experiencing IBD [3]. IBD is a group of chronic inflammatory conditions comprising Crohn’s disease (CD) and ulcerative colitis (UC), with increasing incidence worldwide, which mainly affect the gastrointestinal tract [3,7]. Disease activity varies during the course of disease, as both the extent of inflammation and symptoms change over time [3]. Abdominal pain, bloating, and disturbed bowel patterns are characteristics shared by IBS patients and often referred to as “IBS-like.” Indeed, abdominal pain in IBD has often been ascribed to co-existing IBS, where pain is considered a cardinal feature, and the overlap between IBD and IBS has proven difficult to extricate [2].

The specific mechanisms underlying the pathophysiology of chronic visceral pain still remain to be fully elucidated, however, it has been ascertained that visceral hypersensitivity can occur due to sensitization of primary sensory afferents innervating the viscera, hyperexcitability of spinal ascending neurons (central sensitization) receiving synaptic input from the viscera, and dysregulation of descending pathways modulating spinal nociception [2,45]. Recent theories suggest that neuronal plasticity and broad alterations occurring along the gut–brain axis underlie the development of chronic visceral pain [5]. For instance, there is significant evidence suggesting that permanent alterations in the stress responsive regions and in the descending modulatory system contribute to the development of chronic visceral hypersensitivity [46]. Indeed, it is well established that the stress-induced activation of the hypothalamic–pituitary adrenal (HPA) axis, via the hormonal cascade represented by corticotrophin-releasing factor (CRF), adenocorticotrophin hormone (ACTH), and cortisol, has an important role in the regulation of several functions of the brain–gut axis, including modulation of visceral sensation [47]. Adaptation of extrinsic and intrinsic primary afferents to respond to alterations in the enteric microenvironment contribute to gastrointestinal dysfunction and may also sustain increased visceral nociception [6,35,48,49]. Increased permeability, enhanced interactions with gut saprophytic and pathogen microorganism, inflammation, and neuroimmune crosstalk are just some of the mechanisms occurring within the enteric microenvironment that can result in afferent sensitization [35,37,48]. Accumulating evidence links glial cells with the development and maintenance of chronic pain. Astrocytes and microglia in the CNS and satellite glia in DRGs contribute to development of chronic pain via alterations of glial networks and reactive gliosis. In addition, enteric glia cells, a unique type of peripheral glia found within the ENS, which share many features with CNS astrocytes, may modify visceral perception through interactions with neurons and immune cells [50]. Such rearrangements and vulnerability of central and peripheral nociceptive pathways after exposure to environmental insults and noxious and inflammatory stimuli may increase in critical periods of the lifetime, such as during early life, and predispose to the onset of chronic GI disorders, such as IBS and IBD, later in life [35,51,52,53]. Taking into account the complex and only partially known mechanisms of altered visceral pain perception, the development of novel therapeutic symptomatic approaches is compelling [41]. Currently, conventional analgesics prescribed for somatic pain treatment, such as opioids and NSAIDs, are also indicated for both acute and chronic forms of visceral pain, although it is now recognized that visceral and somatic pains present different pathophysiological phenotypes, and thus required different therapeutic approaches [2,3]. In addition, centrally acting benzodiazepines and antidepressants or peripherally acting antispasmodics are also indicated for visceral hypersensitivity [1,3]. However, all these drugs have been associated with unsatisfactory results in terms of both pain control and side effects, like addiction, analgesic tolerance, and constipation [1,3]. In this view, development of new analgesics targeting visceral hypersensitivity is an ongoing challenge for basic and clinical research. In view of recent advances in the understanding of the pathogenesis of chronic abdominal pain, novel therapeutic agents for the management of visceral hypersensitivity comprise compounds able (1) to alter the gut–brain axis, (2) to modulate local and systemic neuroimmune response, or (3) to suppress neuronal activation and signaling from the gut to the CNS by modulating neuronal excitability of primary afferent neurons [1,41]. In this latter regard, accumulating evidence has shown that several receptors and ion channels may represent promising targets for visceral pain relief [1,41].

## 4. Chronic Abdominal Visceral Pain: Focus on Marine Toxins as Possible Therapeutic Tools 

**Figure 2:**
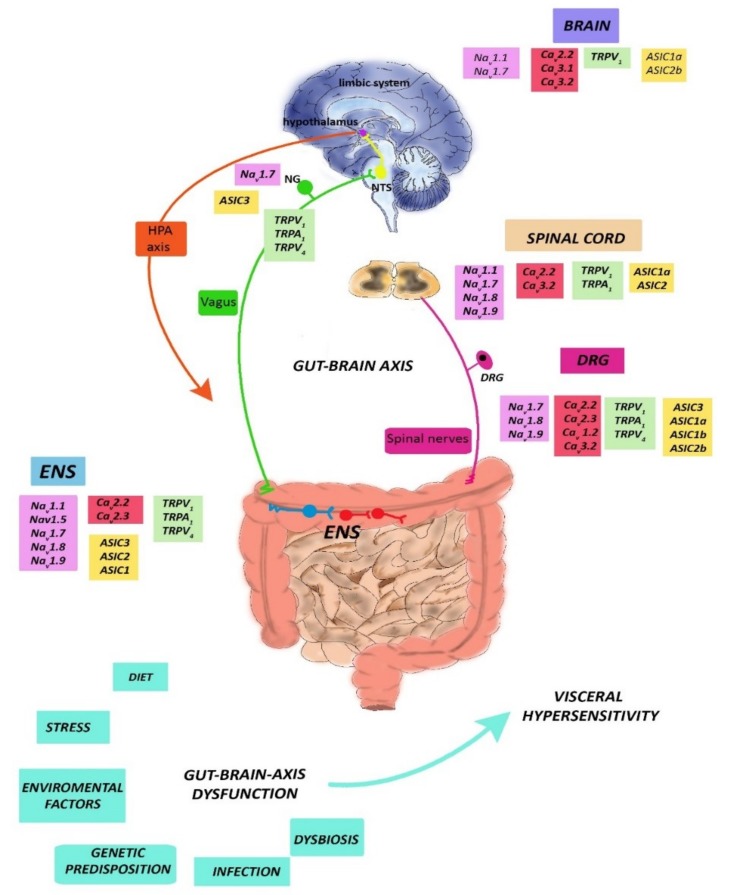
Distribution of the main ion channels involved in visceral pain along the gut–brain axis. Different factors, including environmental changes, stress, diet, previous infection, and alterations of gut microbial flora may alter the stability of the gut–brain communication axis underlying the development of hypersensitivity associated with IBS and IBD. Subunits of voltage-gated sodium channels (Nav) and of voltage-gated calcium channels (Cav), TRP channels (TRPV1, TRPV4, TRPA1), and acid sensing ion channels (ASIC1, ASIC 2, and ASIC3) located in the ENS, in vagal and spinal primary afferent fibers, in intermediate stations of the spinal cord, and in the brain stem and higher brain centers participate in modulation of visceral pain perception. Some of these molecular players are targets for marine toxins, as illustrated in the text and Table 1. Abbreviations: NG, nodose ganglion; DRG, dorsal root ganglion; NTS, nucleus of the solitary tract.

Neurotoxins, including marine toxins, represent biologically active molecules able to interact with high selectivity and potency with a wide range of ion channels and receptors involved in pain generation [8]. Neurotoxins can be used to investigate the properties of the functionally essential domains of pain-related ion channels and receptors, improving the knowledge of their pathophysiological properties [11,54]. From this perspective, the use of neurotoxins may aid to gain important information about the incompletely understood mechanisms underlying visceral pain perception, eventually leading to the discovery of new therapeutic compounds. In addition, neurotoxins may represent lead compounds for the discovery of new drugs in the field of analgesia [54]. In the following paragraphs, we provide an overview on the more recent experimental evidence showing the ability of marine toxins to interact with some of the most important visceral pain molecular modulators, comprising VGSCs, VGCCs, TRPs, and ASIC channels (Figure 2 and Table 1).

### 4.1. Toxins Active at VGSCs

#### 4.1.1. VGSCs

A large and growing body of evidence suggests that VGSCs play a crucial role in the development and modulation of gut-associated visceral pain [66]. VGSCs are responsible for the initiation and propagation of the action potential by enhancing Na^+^ permeability in excitable cell membranes. VGSCs are composed of an α subunit, which forms the transmembrane pore, and at least one accessory β subunit [67]. The α subunit is a relatively large protein (260 kDa) composed of four domains (DI–IV) characterized by six membrane-spanning α-helical segments (S1–S6); these molecular components are sensitive to membrane voltage and are critical to generation and conduction of electrical signals within neurons and skeletal, cardiac, and smooth muscle tissue [67]. The β subunits have a modulatory role by regulating the kinetics and voltage dependence of Na^+^ channel gating and interacting with cell adhesion molecules and extracellular matrix proteins [67]. Genomic sequencing studies indicate that there are nine separate human genes (SCN1A–SCN6A and SCN9A–SCN11A) encoding nine subtypes the of α-subunit, respectively (Na_v_1.1–Na_v_ 1.9), and four genes (SCN1B–SCN4B) encoding five β-subunit isoforms (β1, β1B, β2, β3, and β4) [68,69]. Variations in VGSC sensitivity to different pharmacological mediators, especially to naturally existing toxins, have proven useful in defining the role of each channel subtype, including visceral pain perception [66]. VGSCs can be differentiated based upon their functional response to the venom produced by the puffer fish, tetrodotoxin (TTX). TTX directly binds to the ion selective pore forming subunit α [70]. Na_v_1.1, Na_v_1.2, Na_v_1.3, Na_v_1.4, Na_v_1.6, and Na_v_1.7 are blocked by nanomolar TTX concentrations, and they are described as being “tetrodotoxin-sensitive” (TTX-S), whereas Na_v_1.5, Na_v_1.8, and Na_v_1.9 require much higher concentrations (micromolar) of TTX to be inactivated, and they are considered “tetrodotoxin-resistant” (TTX-R) [71]. The Na_v_1.1, Na_v_1.3, and Na_v_1.6 through Na_v_1.9 channels are involved in nociceptive functions under normal and pathological conditions [72]. The different Na_v_ isoforms have distinct functional properties according to their distribution within different tissues. Na_v_1.1, Na_v_1.2, and Na_v_1.6 are abundantly expressed in the CNS, whereas Na_v_ 1.7, Na_v_ 1.8, and Na_v_ 1.9 are mainly expressed in the peripheral nervous system [73]. Na_v_1.1, Na_v_1.6, Na_v_1.7, Na_v_1.8, and Na_v_1.9 types are expressed in DRG, more than in any other neuronal cell type [72]. Na_v_1.4 and Na_v_1.5 are mainly located on skeletal and cardiac myocytes, while Na_v_1.3 channels are involved in neuronal development and adaptation to nerve damage and may underlie hyperexcitability and ectopic firing after spinal cord or nerve sciatic injury [74,75,76]. From a mechanistic viewpoint, Na_v_ channels regulate nociception by reducing the activation threshold and increasing current density in sensory neurons. A dysregulated expression of these channels may, therefore, contribute to the development and maintenance of pain states [72]. Na_v_1.7, Na_v_1.8, and Na_v_1.9, due to their peripheral location and significant roles in transducing pain stimuli in humans, including altered visceral pain, represent promising targets for the development of analgesic drugs for the treatment of gut-associated pain disorders [66,77]. Upregulation of Na_v_1.7, Na_v_1.8, and Na_v_1.9 channels in DRG and sensory and myenteric neurons has been found in animal models of intestinal inflammatory pain, and further confirmed by knockout studies in mice [78,79]. Increased density of Na_v_1.7 immunoreactive nerve fibers was found in biopsy samples of patients with idiopathic rectal hypersensitivity compared to healthy controls [80]. In rat lower lumbar DRG neurons, Na_v_1.7 mRNA expression increased after induction of both chronic stress and experimental colitis models [81,82]. A Na_v_1.7 specific antagonist, TRTX-Hhn1b, could dose-dependently reverse hyperalgesia after induction of intestinal inflammation in mice [83]. Na_v_1.8 and Na_v_1.9 also have a pivotal role in visceral pain perception from the gut, and changes in their expression have been related to nociceptor activity in different gut disease states, such as inflammation, diabetic intestinal neuropathy, stress-induced visceral hypersensitivity, and bowel obstruction [66,79]. Interestingly, in rodent models, reduced abdominal gut pain and hyperalgesia were obtained after complete genetic deletion of Na_v_ 1.8 or Na_v_ 1.9 [84,85,86]. Furthermore, selective pharmacological blockade of Na_v_1.8 sodium channels with A-803476 produced significant antinociception in rat models of neuropathic and inflammatory pain, although no evidence of the efficacy of this treatment in humans has yet been proven [87]. Na_v_1.5, which is localized on intestinal primary afferents, myenteric neurons, and on non-neuronal cells, such as intestinal smooth muscle cells and interstitial cells of Cajal, has been demonstrated to be involved in the pathogenesis of IBS and other functional GI disorders [88]. IBS and functional dyspepsia patients carry mutations in SCN5a, the gene encoding for Na_v_1.5, which are prevalently loss-of-function mutations and are mostly associated with the constipation-predominant IBS subtype [89,90]. However, a relationship between this mutation and the development of IBS-related visceral hypersensitivity has not yet been proven [66].

#### 4.1.2. Tetrodotoxin and Saxitoxin

Tetrodotoxin (TTX) and saxitoxin (STX) are two guanidinium toxins produced by different marine species that, despite their widespread geographical distribution, share common functional properties acting on the same molecular targets. Both toxins are highly selective blockers of VGSCs, occluding the outer pore of the channel by binding to the neurotoxin site 1 located on VGSC α-subunit [91]. This action impedes influx of sodium ions into the cell, consequently inhibiting cell membrane depolarization and action potential propagation in excitable tissues [70].

Tetrodotoxin (TTX) is one of the best-characterized marine toxins due to its involvement in food poisoning, having been responsible for many intoxications and fatalities, including in recent decades. Initially detected in pufferfish, a fish of the family *Tetrodontidae*, TTX is also present in a variety of marine and some terrestrial organisms, which use the toxin as a defense against predators, and in marine algae [92,93]. Although the exact sources generating the neurotoxin are unknown, there are several hints suggesting that TTX, accumulating within living organisms, is acquired from the food chain. Recent studies indicate endosymbiotic bacteria as a primary source able to produce TTX [92]. The toxin is one of the most powerful neurotoxins, with more than a thousand-fold higher toxicity than cyanide in humans and no available antidotes [93,94]. Clinical manifestations of intoxication include nausea, vomiting, abdominal pain, diarrhea, headache, facial paraesthesia, itching and skin burning, and muscular weakness, followed, in severe cases, by muscle flaccid paralysis, convulsions, and respiratory and heart failure [94]. In view of the dissimilar sensitivities of different Na_v_ isoforms to the toxin and to their distinct distribution profile, the response of different excitable tissues to TTX depends on the discrete VGSC isoforms present in different cell types [71,95]. Numerous studies suggest that TTX-sensitive subtype VGSCs are involved in the modulation of normal and pathological pain perception. From this perspective, TTX may have a potential antinociceptive role, and the neurotoxin has been investigated in different animal models of inflammatory, neuropathic, and visceral pain [55,96,97]. Furthermore, the lack of affinity for Na_v_1.5 channels, which are mainly expressed in the cardiac tissue, confers a rather safe profile to the toxin, which, among Na_v_ blocking molecules, is one of the more attractive for analgesic purposes, as also demonstrated by numerous on-going patented studies [98]. Despite the key role of TTX-sensitive Na_v_1.7 channels in pain perception, a limited number of preclinical studies have been conducted to date on the ability of the toxin to relieve acute pain, and available data suggest that TTX has little impact in acute pain responses [71]. More promising results were obtained regarding the efficacy of TTX in the treatment of neurogenic chronic pain induced by experimental inflammation and, most of all, of neuropathic pain, where TTX has been demonstrated to significantly reduce hyperalgesia, mechanical allodynia, and spontaneous development of afferent activity caused by peripheral nerve injury [99,100,101,102,103]. Significant data on the analgesic efficacy of TTX have also been obtained in animal models of chemotherapy-induced peripheral neuropathy, which represents the major dose-limiting side effect of many chemotherapeutic drugs. In this context, clinical trials have yielded promising results for the treatment of moderate to severe cancer pain [71]. In a recent Phase III multicentre, randomized, double-blind, placebo-controlled trial, subcutaneous administration of TTX twice daily for four consecutive days gave promising analgesic effects for the treatment of neuropathic pain and inadequately controlled cancer-related pain (NCT00725114) [104]. In addition, this treatment proved to have a high tolerability, since adverse events were generally mild to moderate and transient, characterized in general by nausea, dizziness, and oral numbness or tingling. Although the study was stopped prematurely before reaching the planned sample size, and is thus considered unpowered, it provided further evidence, with respect to earlier trials, for the safety and efficacy of TTX in the treatment of neuropathic and cancer pain [105,106].

TTX has also been proven to be efficacious in different experimental models of visceral pain. In the acetic acid writhing test in mice, subcutaneous administration of TTX dose-dependently reduced the number of abdominal contractions. Interestingly, morphine attenuated this pain response, but with a lower safety profile than TTX [56]. In a recent study carried out in mouse colon, chemical stimulation with capsaicin and mustard oil were used to evaluate efficacy of TTX in attenuating viscero-specific pain responses [55]. In this study, subcutaneous administration of TTX dose-dependently inhibited the number of pain-related behaviors after application of both irritant stimuli, but was able to reverse only capsaicin-induced mechanical hyperalgesia, possibly owing to the differential noxious pathways and downstream inflammatory/irritant mechanisms activated by the two chemicals [55]. TTX reduced capsaicin and mustard oil colonic stimulation pain response in conditional Na_v_1.7 knockout mice to the same extent as littermate controls, suggesting the participation of TTX-sensitive Na_v_ subtypes other than Na_v_1.7 [107]. Interestingly, in a mouse model of chronic visceral mechanical hypersensitivity, which represents a model of abdominal pain associated with IBS, functional upregulation of TTX-sensitive Na_v_1.1 developed in a subset of high-threshold mechanosensitive colonic fibers [108]. Thus, from a translational viewpoint, upregulation of Na_v_1.1 may also sustain development of chronic visceral hypersensitivity in IBS patients. Furthermore, another TTX-sensitive Na_v_ channel, Na_v_1.3, which is generally undetectable in adult DRG, may be expressed during pathophysiological conditions, i.e., during chronic pain disorders. Although there have been no indications as to the modulation of Na_v_1.3 expression after induction of viscero-specific pain responses, overexpression of this channel has been demonstrated in DRG neurons, dorsal spinal cord, and hypothalamus of rodent models after induction of inflammatory and neuropathic pain [75,76]. Taken together, these reports suggest that TTX may represent a promising and useful instrument to treat visceral pain, although more studies are needed to better clarify the molecular mechanisms underpinning its potential analgesic efficacy for gut visceral pain treatment. An important limitation for the clinical use of TTX as an analgesic is represented by its potential toxicity, which, however, in view of the toxin’s poor permeability through the blood–brain barrier, does not involve CNS functions [105,109]. Most side effects, indeed, comprise peripheral inhibition of motor and sensory functions [105,109]. Another crucial factor for the potential therapeutic applications of TTX is represented by the stringent relationship between its analgesic efficacy and its ability to permeate target tissues. The latter can be improved by administration of additional drugs, such as vasoconstrictors and local anesthetics, or with targeted delivery systems such as microparticles and liposomes conjugated with nanogolds [98]. In addition, structural modifications of the alkaloid may ameliorate its therapeutic properties, leading to new drug analogues. This effort has been addressed to obtain STX analogues, which, as with TTX, may have potential pharmaceutical analgesic properties. STX was discovered in 1789 and is also known as a “paralytic shellfish toxin” (PST), from a severe intoxication called “paralytic shellfish poisoning.” STX and related derivates are alkaloids naturally produced by three genera of marine dinoflagellates and freshwater or brackish cyanobacteria during algal bloom events [110]. Several species within the cyanobacterial genera, *Cylindrospermopsis*, *Dolichospermum*, *Aphanizomenon*, *Planktothrix*, and *Lyngbya* synthesize PSTs [111]. There are at least 57 analogs of STX, including those directly synthesized by dinoflagellates and those biotransformed in other species [112]. Voltage-clamp studies have shown that STXs may target and block Na^+^ conductance associated with cell excitation [113,114] and, to a minor extent, also potassium and calcium channels [115,116]. Analogously to TTX, STX, by interacting with subunit 1 of VGSCs, has analgesic properties and induces anesthesia or extends the anesthetic effect of local anesthetics in combined therapies [117], however, the toxin’s systemic toxicity prevents its clinical application. Microencapsulation within liposomes has been proposed for ameliorating the toxin safety for the treatment of joint pain and intractable localized pain [118]. For example, 18 day liposome injection of STX was described to produce a prolonged nerve blockade contrasting allodynia in a rat model of neuropathic pain without any myo- or neurotoxicity [119]. Contrary to the parent compound, purified STX analogs, differing slightly from the parent molecule by one or a combination of substituted sulfate, carbamoyl, hydroxy, or benzoate groups, have been used as therapeutic agents with considerable success in recent medical trials [91,112,120]. In the gastrointestinal tract, a mixture of gonyautoxin 2 and 3 epimers (GTX 2/3) was applied during acute and chronic anal fissures to safely produce flaccid paralysis of the anal muscle, thereby accelerating tissue healing [121]. Local infiltration with GTX 2/3 mixture was also effective, as a substitute for opioids, for the pain treatment of total knee arthroplasty [122]. In rats, an N-1 hydroxylated STX analog, neoSTX, displayed efficacy for both acute and chronic pain treatment via different administration routes, also displaying a safe profile without major toxic symptoms [123]. A double-blind, placebo-controlled, randomized study carried out on 10 healthy volunteers showed the analgesic efficacy of neoSTX on different types of somatic pain without major adverse effects [124]. In a successive Phase I clinical trial carried out as a double-blind, randomized, controlled study on 84 healthy male volunteers, a subcutaneous dose of NeoSTX, administered alone and in combination with bupivacaine with or without epinephrine, displayed a tolerable side effect profile and showed promising efficacy for prolonged local anesthesia [125]. A few studies are also available, with promising results, for the treatment of visceral pain disorders with neoSTX. For instance, local infiltration of neoSTX was efficacious in attenuating bladder pain syndrome [126]. Moreover, in a pilot clinical study by Rodriguez-Navarro and colleagues, neoSTX administered by intrasphincteric injection during endoscopy for the treatment of achalasia, a gut motility disorder resulting from a failure of the lower esophageal sphincter that causes dysphagia and chest pain, induced a rapid and long amelioration of the symptoms [127]. 

### 4.2. Toxins Active at VGCC

#### 4.2.1. VGCCs 

VGCCs are key transducers of membrane potential changes into intracellular Ca^++^ transients that initiate many physiological events, such as secretion, regulation of gene expression, smooth muscle contraction, integration of synaptic transmission in neurons, and firing of action potentials in rhythmically firing cells such as cardiac myocytes and thalamic neurons [128]. VGCCs in these different cell types activate on membrane depolarization and mediate influx of Ca^++^ in response to action potentials and subthreshold depolarizing signals [128]. Nine different types of VGCC, clustered into three major families, Ca_v_1, Ca_v_2, and Ca_v_ 3, have been identified at a neuronal level in vertebrates [129]. Ca_v_1 and Ca_v_2 belong to the superfamily of high-voltage-activated (HVA) calcium channels that require large membrane depolarizations to open, while Ca_v_3 are low-voltage-activated (LVA) calcium channels, which open in response to smaller depolarizations [130]. Within this classification, N-type (Ca_v_ 2.2), L-type (Ca_v_ 1.2, Ca_v_ 1.3, Ca_v_ 1.4), R-type (Ca_v_ 2.3), and P/Q- type (Ca_v_ 2.1) belong to the HVA class, and the T-type (Ca_v_ 3.1, Ca_v_ 3.2, Ca_v_ 3.3) to the LVA class [129,131]. Neuronal HVA calcium channels are heteromultimeric channels, comprising principally Ca_v_α1, Ca_v_β, and Ca_v_α2δ subunits that coassemble with 1:1:1 stoichiometry into a functional calcium channel complex, with the Ca_v_α1 representing the pore-forming unit, capable of producing calcium currents even in the absence of accessory subunits [130]. VGCCs may exhibit different localization and function in neuronal cells. N-type and P/Q type are expressed on the presynaptic nerve terminal, where they control neurotransmitter release, whereas T type channels are mainly expressed at dendritic sites and cell bodies, contributing to fine-tuning neuronal excitability [132,133]. VGCCs have unique pharmacological profiles, which have long been used to identify different types of calcium currents: Ca_v_2.2 channels are blocked selectively by the cone snail ω-conotoxin GVIA, MVIIA, and α-conotoxin; P/Q-type channels by the spider toxin ω-agatoxin IVA; R-type channels are selectively blocked by peptide SNX-482, derived from the tarantula *Hysterocrates gigas*; and L-type calcium channels by a wide range of dihydropyridines. T-type and R-type channels can be inhibited by nickel ions [132].

VGCCs channels play a fundamental role in the transmission and processing of pain related information both along primary afferent pathways and in higher brain centers [129]. VGCCs modulate visceral nociceptive signaling from the gut to the spinal cord through DRG neurons, involved in development of chronic visceral pain and hypersensitivity [134]. N-type channels participate in transducing nociceptive signals and are highly expressed in spinal dorsal horn neurons, in the cell soma of DRGs, and in the synaptic connection between spinal dorsal horn neurons and central terminals [135,136]. The ability of N-type channels to exert a key role in pain processing is suggested by their co-localization with nociceptive neurotransmitters, such as substance P, CGRP, and glutamate, and by their upregulation in spinal dorsal horn during maintenance of pain states after nerve injury [137,138]. In the guinea-pig small intestine ENS, N (α1B), and R (α1A) channels are the predominant VGCCs expressed in the cell bodies of IPANs, where the N-type current contributes to a significant extent to the inward Ca^2+^ current associated with the propagation of action potentials [139]. Rat colonic specific DRG neurons also express Ca_v_1.2 L-type and R-type channels, the levels and function of which were significantly up-regulated following experimentally-induced colitis. Such up-regulation was associated with increased visceral sensitivity to mechanical stimuli and was reduced by intrathecal administration of the specific inhibitors, nimodipine and SNX482, respectively [140]. As regards L-type channels, the nociceptive action of Ca_v_1.2 does not only depend on neuronal mechanisms, but also on the channel’s ability to act on intestinal smooth muscle, where the channel controls both spontaneous and electrically evoked contractions [141]. In a model of IBS, increased transcription of Ca_v_1.2 has been related to smooth muscle hyperactivity and acceleration of colonic transit [142]. T-type channels also seem to play a fundamental role in the development of chronic visceral pain [143,144]. T-type channels are expressed along the pain axis in DRG, superficial spinal dorsal horn neurons, and on thalamic neurons [145]. Ca_v_3.2 T-type channels were upregulated in DRG neurons, spinal cord, and thalamus following visceral inflammation [146,147]. Analogously, genetic deletion of Ca_v_3.1 channel was associated with increased visceral pain, which correlated to the channel function in the thalamus [148]. Activation of T-type calcium channel subtype on primary spinal visceral afferents has been associated with IBS-like symptoms in animal models. In a rat model of IBS, enhanced expression of a T-type channel, Ca_v_3.2, coincided with development of hypersensitivity to colorectal distension, proposing this VGCC as a potential target for the treatment of IBS-associated chronic pain [143]. 

#### 4.2.2. ω-Conotoxin

ω-conotoxins are a family of disulphide-rich basic peptides of 8–31 amino acids, with a fold-loop structure, produced by predatory mollusks from the genus *Conus*. The venom from each of the estimated 500 species of cone snails may contain 50–200 distinct biologically active peptides [149]. Among the best characterized are ω-conotoxin GVIA, produced by *Conus geographus*, ω-conotoxin CVID, isolated from *Conus catus*, and ω-conotoxin MVIIA, produced by *Conus magus*. ω-conotoxins GVIA, MVIIA, and CVID have been extensively used to elucidate the distribution and physiological properties of VGCCs [150]. Indeed, ω-conotoxins are the most selective inhibitors of N-type VGCCs along the neuronal pain axis, representing important molecules for the discovery of new analgesic drugs [151]. In this regard, ziconotide, also known as SNX-111, and, most recently, Prialt^®^, a synthetic analog of ω-conotoxin MVIIA, produced a significant analgesic effect in different animal models, including chronic neuropathic and inflammatory pain, which, unlike opoids, was not associated with development of tolerance [152,153,154]. Based on its efficacy and tolerability in preclinical studies, ziconotide was moved to human trials in the quest for alternatives to opioid-based analgesic treatments, which may induce important side effects including hyperalgesia, tolerance, addiction, and endocrinological and neurological disorders [155]. Ziconotide was approved by the FDA in 2004 as an intrathecal analgesic for the treatment of severe refractory chronic pain [156]. Randomized control trials on the efficacy of intrathecal ziconotide monotherapy demonstrated good efficacy in patients affected by chronic pain, AIDS, or cancer [157]. To synergistically increase pain relief, compared to monotherapy, ziconotide has also been used in combination with opioid agents in the treatment of refractory chronic and cancer pain [158]. Clinically, ziconotide is, however, considered as a “last resort,” owing to the relatively costly and invasive procedure of intrathecal administration, as well as the safety profile [157]. Intrathecal administration of ziconotide is associated with infusion-rate-dependent adverse effects, including nausea and/or vomiting, dizziness, confusion, urinary retention, somnolence, and more debilitating ataxia and psychosis [159]. In spite of these shortcomings, the peptide has recently been considered a first line analgesic for the treatment of neuropathic and nociceptive pain [160]. The mechanism of action of ziconotide is based on the ability of the compound to inhibit the α_1B subunit of pre-synaptic N-type channels in the central terminals of nociceptors, reducing the release of pro-nociceptive neurotransmitters, and thereby disrupting the transmission of pain signals in the spinal cord [134]. Interestingly, ω-conotoxin MVIIA was able to block the release of glutamate in the cerebrospinal fluid in mouse models of gut visceral nociception induced by intraperitoneal injection of acetic acid or intracolonic application of capsaicin [57]. This effect is particularly interesting in view of the key role played by glutamate and excitatory amino acids in the transmission of visceral sensory information to dorsal horn neurons and thereby to higher CNS centers of gut visceral pain elaboration [161,162,163]. However, the considerable side effects limit ziconotide clinical use, and other ω-conotoxins, such as CVID (AM336, Leconotide, CNSB004), have been synthesized with an improved safety profile, especially with lower cardiovascular effects [164,165]. The more favorable action of CVID compared to MVIIA can be explained on the basis of the higher selectivity for a N-type Ca_v_2.2 calcium channel variant in preganglionic nerve terminals, better in vitro reversibility, and faster onset/offset of action [166]. CVID reduced hyperalgesia in a rat model of neuropathic pain after intravenous administration [167]. Based on preclinical studies on the efficacy at relieving cancer and diabetic neuropathic pain after intravenous CVID, the drug has been proposed as an intravenous analgesic to be co-administered with opioids and non-opioid analgesics [165,167,168]. However, in spite of the promising preclinical results, clinical PhaseI/II trials on the efficacy of CVID to reduce cancer pain refractory to standard analgesic therapy have been interrupted owing to the low safety profile at higher intrathecal doses, resulting in nausea, disorientation, dizziness, and hallucinations, similarly to other conotoxins, and to the inability to translate the potency ratio from preclinical studies to human practice [165]. More recently synthesized ω-conotoxins, acting as potent N-type VGCC selective blockers, comprise CVIE and CVIF from *Conus catus* and ω-conotoxin FVIA, isolated from *Conus fulmen* [169,170]. Preliminary investigations have been carried out with promising results for the treatment of different nociceptive behaviors, including neuropathic pain and mechanical and thermal allodynia. However, further studies are needed to clarify the therapeutic potential of these compounds, and there are no indications for their potential efficacy in the treatment of gut visceral pain syndromes.

#### 4.2.3. α-Conotoxin

The family of α-conotoxins (α-CTxs) has produced promising results supporting the possibility to develop therapeutically valuable analgesics. α-CTxs are a family of relatively short, disulfide-rich peptides (12–20 amino acids), and, to date, 45 α-conotoxins from 23 different cone snail species have been recognized with varying degrees of subtype selectivity [171]. α-CTxs have different molecular targets, principally including muscle and neuronal nicotinic acetylcholine receptors (nAChR), N-type VGCCs, and gamma aminobutyric acid B (GABA_B_) receptors [172,173,174]. α-conotoxins Vc1.1, RgIA, and PeIA are potent antagonists at nAChRs [172]. To date, 16 neuronal nAChR subunits have been recognized in the human genome (α1–α7, α9, α10, β1–β4, δ, ε, and γ), and the heterogeneity of their matching confers different functions and sensitivity to ligands at the site located at the N-terminal domain of the receptor [175]. nAChR subunit expression differs according to the tissue, with α1, β1, δ, ε, and γ being located in striated muscle, while α2–α7, α9, α10, and β2–β4 are considered neuronal receptors, although they are also expressed in non-neuronal tissues [176]. Neuronal nAChRs have been explored as targets for pain treatment for about 30 years. Although the initial studies were focused on the α4β2 subtype, recently, α6- and α9-containing receptors have been considered as novel therapeutic targets. In particular, α6β4 subunits were shown to be expressed in DRG, and, together with α3β4 or β2-containing subtypes, have been suggested to participate in pain perception [177]. α9 nAChRs, located on DRG neurons, have been shown to play a role in chronic pain perception in several models of neuropathic pain and after a traumatic nerve injury [178,179]. In addition, accumulating evidence suggests that agonists and modulators of α7 nAChRs may be promising in the treatment of pain conditions, including visceral pain [180,181]. However, despite these efforts, no nAChR modulator has yet been approved as an analgesic, principally owing to safety concerns around possible cardiovascular and gastrointestinal side effects [182].

α-CTxs display different selectivity for nAChRs, for example, α-conotoxins ArIB and PnIA bind to α7 and α3β2 nAChRs [183], PelA potently blocks α9α10, α3β2, and α6-containing nAChRs, whereas RgIA and its analogues and Vc1.1 selectively block α9α10 nAChRs [184,185]. In particular, α-conotoxin Vc1.1 was the first cloned from mRNA of the venom ducts from the tropical marine snail, *Conus victoriae* [186], and diverges from the native peptide Vc1a in the lack of two post-translationally modified residues [187]. The synthetic form Vc1.1 along with RgIA, cloned from *Conus regius*, was able to relieve allodynia and accelerate the functional recovery of injured neurons in a rat model of neuropathic pain [185,188,189]. The molecular mechanisms involved in the analgesic effects of α-conotoxins are controversial. Although the α9α10 nAChR subtype antagonism has been proposed as a target for these antinociceptive actions, the observation that structural analogues of Vc1.1 selectively binding to α9α10 nAChR failed to inhibit allodynia suggests the involvement of other mechanism/s [190]. This latter hypothesis was confirmed by other studies demonstrating that other α-CTxs, inactive on α9α10 nAChR, and requiring α3β4, α3β2, or α6-containing nAChRs subtypes, such as AuIB and MII, produced potent, long-lasting reversal of allodynia [191,192,193]. Indeed, a further mechanism underlying Vc1.1 and RgIA analgesic action is represented by the indirect inhibition of VGCCs, Ca_v_2.2, and Ca_v_2.3 via G protein coupled GABA_B_ receptor activation [194]. GABA_B_ receptor modulation of N-type VGCCs is largely mediated via voltage-independent intracellular pathway(s), requiring src tyrosine kinase activity, and does not directly involve βγ G protein subunits [194]. GABA, the major inhibitory neurotransmitter in the CNS, plays an important role in antinociception, and its participation in visceral pain is supported by several studies [1]. Indeed, activation of GABA_B_ receptors with baclofen has shown promising results for the treatment of gut visceral pain, by reducing visceromotor responses to colorectal distension [195,196,197]. This effect derives from a direct influence on peripheral sensory inputs, but also from the activation of inhibitory interneurons within the CNS. However, tolerance to the therapeutic effects, the narrow therapeutic index, and side effects of baclofen represent problematic issues for its clinical use, and other mechanisms of GABA_B_ signaling activation, such as positive allosteric modulation, represent possible valid alternatives and are under investigation. Among these modulators, analogues of Vc1.1 showed promising results in both neuropathic and visceral pain [58,192]. In this latter regard, Vc1.1 was able to inhibit murine colonic sensory afferents ex vivo, as well as nociceptive signaling of noxious colorectal distension, with higher efficacy during experimentally induced visceral hypersensitivity [58]. In addition, Vc1.1 reduced human DRG excitability via GABA_B_ receptor activation and downstream inhibition of Ca_v_2.2 and Ca_v_2.3 VGCCs [58]. In view of the promising preclinical findings on its potential analgesic properties, Vc1.1 entered Phase I and Phase II clinical trials for the treatment of neuropathic pain by Metabolic Pharmaceuticals Limited (coded as ACV1) [198]. These studies demonstrated that the molecule was well tolerated with a safe side-effect profile [198]. However, these trials were interrupted in 2007 owing to the limited clinical efficacy of the peptide, which was attributed to its molecular instability, lack of selectivity for GABA_B_ receptor, and lower potency at α9α10 nAChR when translating the action from rodents to humans [58,155,199]. The difference in ligand sensitivity among different species, the susceptibility to degradation, and the lack of selectivity associated with the development of serious side effects represent the major drawbacks for the potential therapeutic use of conotoxins. In this view, several investigations have been carried out to re-engineer the structure of the native peptide in order to obtain molecules with a higher selective binding profile to either nAChRs and/or GABA_B_ receptors, to ameliorate their stability and improve their biopharmaceutical properties [200,201,202]. In particular, a cyclized analogue of Vc1.1, cVc1.1, has shown several benefits over its parent compound Vc1.1, including a higher potency in promoting GABA_B_ receptor-mediated inhibition of Ca_v_2.2 channel, a higher selectivity for GABA_B_ receptor vs α9α10 nAChRs, and a good oral bioavailability [202]. cVc1.1 has recently shown promising results, displaying anti-nociceptive effects and inhibiting visceral pain in a mouse model of chronic visceral hypersensitivity by blocking VGCCs with high potency [59,60]. Likewise, a truncated form of Vc1.1, [Ser3] Vc1.1(1-8), inhibited calcium channel currents in mouse DRG neurons, reducing visceromotor response to colorectal distension [61]. Since these altered visceral sensory functions are characteristic of IBS, these observations open a novel perspective on the potential use of modified α-CTXs for the treatment for chronic visceral pain and IBS.

### 4.3. Toxins Active at TRPs

TRPs are important players of visceral nociception [203]. The TRPs consist of a superfamily of more than 30 structurally related, highly conserved cationic channels acting as signal transducers by altering membrane potential or intracellular calcium concentration [204]. Based on sequence homology, the mammalian TRP channel superfamily is classified into six subfamilies: TRPC (canonical), TRPV (vanilloid), TRPM (melastatin), TRPA (ankyrin), TRPML (mucolipin), and TRPP (polycystic) [204]. TRPs are non-selective cation channels, exhibiting differences in permeability and selectivity, that assemble as tetramers composed of six transmembrane domains, with a pore formed by the hydrophobic region between the fifth and sixth segments [204,205]. TRP channel families A, C, M, and V are expressed on afferent pathways from the viscera, where they are physiologically involved in the regulation of several functions, ranging from thermo-, mechano-, and chemosensors to transducers of other receptors (GPCRs or cytokine receptors) [206]. TRPA1, V1, V4, and M8 have been shown to coexist on the same gut sensory neuron, and may interact in the transduction of nociceptive stimuli [206]. Among these, TRPV1, located predominantly on capsaicin-sensitive sensory neurons, represents one of the more interesting receptor classes involved in inflammation-induced pain as well as in colorectal mechanosensation [207]. The importance of TRPV1 in visceral pain perception during disease states is underscored by the evidence that this TRP is involved not only in lower gastrointestinal diseases, but also in upper gut disorders, such as gastroesophageal reflux disease and pancreatitis [208,209]. Activation of TRPV1 signaling on intrinsic and extrinsic primary afferents leads to transfer of pain information to the CNS, causing the release of different pain mediators, such as substance P, CGRP, and glutamate [210]. Many animal studies point toward activation, and even sensitization, of TRPV1 by various inflammatory mediators [211]. In animal models of intestinal inflammation and in the colon and DRGs of CD patients, locally released nerve growth factor (NGF) and prostaglandins were shown to upregulate TRPV1 with a converging mechanism [212,213,214]. Activation of TRPV1 by protein kinase C and c-AMP-dependent protein kinase phosphorylation underlies facilitation of colorectal afferent neuron sensitization, whereas it is inactivated by phosphatase-mediated dephosphorylation [215]. Indeed, multiple pain mediators (inflammatory cytokines, bradykinin, proteases, and ATP) stimulate TRPV1 via kinase activation [216]. In a rat model of colitis, TRPV1 and TRPA1 synergistically interacted in the modulation of inflammation-induced visceral hypersensitivity [211]. Like TRPV1, TRPA1 is expressed on extrinsic primary afferents from the gut and in the colon ENS [217,218]. Activation of TRPA1 is required for the development of acute pain, and it exerts a crucial role in the transition from acute to chronic pain [219]. In in vivo animal models, TRPA is involved in visceral hypersensitivity and hyperalgesia to colorectal distension [86,220,221]. Upregulation of TRPA1 has been demonstrated in experimental colitis [220]. Accordingly, colitis-induced mechanical hypersensitivity was reduced both in knockout TRPA1 mice and in wild type animals after administration of TRPA1 channel antagonists [220]. TRPV1 and TRPA1 are also located on non-neuronal cells, i.e., vascular smooth muscle cells and inflammatory cells, such as macrophages and T helper cells, where both receptors modulate pro- and anti-inflammatory responses [211]. TRPV1 expression is upregulated not only in patients with IBD in remission, but also in patients with an IBS-like syndrome [222]. Levels of TRPV1 are elevated in patients with rectal hypersensitivity, and their sensory response to rectal distension significantly correlates with TRPV1 levels [212,215]. In neonatal rats, colonic irritation by acetic acid infusion led to persistent colonic sensitivity, which was mainly mediated by TRPV1 upregulation, since intraperitoneal injection of TRPV1 antagonists later in life significantly reduced the hypersensitivity response to distension [223]. Interestingly, intracerebroventricular administration of SB366791, a TRPV1 antagonist, inhibited chemical and inflammatory spontaneous abdominal nociceptive behavioral responses in adult rats, suggesting that TRPV1 is involved in the CNS affective component of visceral nociception [224]. Another TRPV1 antagonist, capsazepine, has been shown to modulate inflammatory-associated visceral pain and disease severity in dextran-sodium-sulfate-induced IBD [222,225]. TRPA1 may also participate in IBS pain symptoms, as suggested by the finding that supernatants from peripheral blood mononuclear cells from patients with diarrhea-predominant IBS caused hypersensitivity of mouse colonic afferent endings via activation of TRPA1 [226]. Overall, these observations have led to a considerable interest in targeting TRP channels, such as TRPV1 and TRPA1, to discover novel therapies for alleviating visceral pain. 

Among different modulators, marine toxins deriving from sea anemones may interact with both TRPV1 and TRPA1, with promising results as potential analgesics [8]. Sea anemones are a source of peptides including phospholipases, Na^+^- and K^+^-channel inhibitors, ASICs inhibitors, and proteinase inhibitors, and may cause severe neurotoxic effects. At the moment, 236 peptide or protein toxins, which can be classified into 15 known families, have been isolated from 45 sea anemone species [227]. APHC1 and APHC3 are polypeptides composed of 56 amino acids extracted from the sea anemone *Heteractis crispa,* with capsaicin-induced current inhibiting properties. Electrophysiological and Ca^++^ imaging investigations revealed that APHCs exert a dose-dependent dual mechanism on capsaicin-sensitive TRPV1 channels, with a potentiating action at low doses and an inhibitory effect at high concentrations [228]. In the mouse, TRPV1 inhibition by both toxins produced analgesic effects on the hot plate and tail flick acute pain models with high potency [229]. In a successive study, APHC1 and APHC3, although showing a moderate efficacy in inhibiting TRPV1 currents in whole cell patch clamp experiments, was proven to be efficacious in reducing pain related responses both in tests directly associated with TRPV1 activation (capsaicin, noxious thermal stimuli, thermal hyperalgesia) and in general models of pain, including the visceral pain model of acetic-acid -induced abdominal constriction in mice [62]. Interestingly, APHC1 and APHC3 were also shown to modulate bladder contractility in a rat model of diabetic cystopathy by a mechanism involving decreased TRPV1-dependent release of tachykinins from bladder afferents and direct suppression of tachykinin degradation [230]. These latter observations suggest that both toxins may be considered effective modulators of disorders associated with development of visceral pain. 

A further group of sea anemone toxins modulating TRPA1 channels is represented by τ-Anm toxins such as Ms 9a-1, a Cys-rich peptide, recently extracted from the venom of *Metridium senile*. Opposite to APHC1 and APHC3, Ms 9a-1 represents a positive modulator of TRPA1 [231]. Exposure of CHO cell cultures, transfected with recombinant TRPA1, to peptide Ms 9a-1 potentiated the response to AITC (TRPA1 agonist), behaving as a positive modulator of the receptor. However, the toxin did not cause pain or thermal hyperalgesia when injected into the hind paw of mice. Furthermore, intravenous injection of Ms 9a-1 produced a significant decrease in the nociceptive and inflammatory response and reversed complete Freund’s adjuvant-induced inflammation and thermal hyperalgesia. The authors suggest that the significant analgesic effect of the toxin is due to receptor desensitization induced by the agonist properties of Ms 9a-1. Although no studies are available, at the moment, on the analgesic properties of Ms 9a-1 in the modulation of visceral pain, this may represent a novel mechanism in order to selectively deregulate active TRPA, ameliorating the safety profile of molecules targeting TRPA1 receptors [232].

### 4.4. Toxins Acting at ASICs

ASICs are sodium-gated ion channels member of the epithelial/degenerin Na^+^ channel family, acting as “chemoelectrical transducers” by sensing the decrease of pH in the extracellular environment [233]. Six ASIC subunits have been characterized to date, which are encoded by four distinct genes (ASIC1-4), since ASIC1 and ASIC2 have been demonstrated to produce two functional splice variants (ASIC1a, ASIC1b, and ASIC2a, ASIC2b, respectively) [233]. Each ASIC subunit consists of short intracellular N- and C-terminals, two transmembrane domains (TM1 and TM2), and a large extracellular domain [234]. ASIC subunits can assemble as heteromeric or homomeric trimers to produce functional channels [235]. ASICs are directly gated by protons during tissue acidosis occurring as a consequence of ischemia, trauma, tumor, and surgery, in particular when the tissue pH value is below 7, and represent the major sensor of extracellular pH changes in nociceptive pathways [236]. ASIC activation by acidic pH induces membrane depolarization and action potentials firing in neuronal pain pathways involved in inflammatory pain and in different chronic pain conditions [8,235]. Functionally active ASIC1–ASIC4 channels have been localized in neurons from rat DRGs, and several findings show that an acidic environment is a potent stimulus for primary afferent neurons, inducing fast currents in DRG neurons [237]. In addition, immunohistochemistry studies revealed colocalization of ASICs with CGRP and substance P in small capsaicin-sensitive nociceptive neurons [238]. In the gastrointestinal tract, ASICs have been detected in extrinsic primary afferent neurons originating from the DRGs and NG [239]. Different functional ASICs are present as homomultimers, mainly represented by ASIC1a and ASIC3, or in heteromultimer combinations of different subunits, in glossopharyngeal, vagal, and spinal afferent neurons innervating the gut and other visceral organs (for a more detailed description of ASIC distribution in the gastrointestinal tract see the review by Holzer [239]. In particular, ASIC3, which represents the most important proton^-^ sensitive channel involved in modulation of moderate- to high-intensity pain sensation, has been almost exclusively localized to primary afferent neurons [240,241,242]. Interestingly, retrograde tracing studies have shown that the majority of the NG and DRG neurons projecting to the rat stomach express ASIC3-like immunoreactivity [243]. Accordingly, the soma of a high percentage of thoracolumbar mouse DRGs projecting to the mouse colon stained for ASIC3 and, to a minor extent, for ASIC2 and ASIC1 [244]. Proton activation of ASICs has been implicated in gastritis, peptic ulceration, and other gastrointestinal disorders associated with altered pain perception, suggesting that modulation of these channels may have potential implications for the relief of visceral gut pain [239,245]. In the upper gastrointestinal tract, ASIC3 has been suggested to play a major role in the development of inflammatory hypersensitivity to gastric acid, occurring during gastritis and peptic ulcer disease, both associated with painful symptoms [246]. In the lower part of the gut, ASIC activation correlates with development of hypersensitivity to colorectal distension, indicating that, analogously to TRP, ASIC may be affected by different stimuli, including mechanical stimuli [247,248,249]. In particular, in a mouse model of zymosan-induced sensitization of colonic mechanoreceptors, both ASIC3 and TRPV1 participated in development of chronic hypersensitivity to colorectal distension in the absence of inflammation, suggesting that both ASIC3 and TRPV1 may contribute to non-inflammatory visceral hypersensitivity, typical of IBS [249]. Intracolonic administration of butyrate in rats induced hyperresponsiveness to colorectal distension, associated with an upregulation of ASIC1a in colonic DRG neurons and in the spinal cord [250,251]. In these experimental conditions, among the different mediators of visceral hypersensitivity, NGF played a key role in crosslinking painful stimuli with spinal upregulation of ASICs and colonic hypersensitivity [252,253]. Interestingly, butyrate-induced upregulation of both ASIC1a and colonic hypersensitivity was prevented by NGF neutralization [251]. These studies suggest that molecules acting at ASICs may represent possible targets for the discovery of drugs active on visceral pain associated with functional gastrointestinal disorders, such as IBS. The sea anemone toxin APETx2 has been shown to modulate pain perception by interacting with ASICs. APETx2 is a 42 amino acid toxin with a defensin-like fold, derived from sea anemone, *Anthopleura elegantissima,* known to affect ASIC3 channels homomers as well as several heteromers of ASIC3 in combination with ASIC1a, ASIC1b, and ASIC2b [254,255]. APETx2 blocks ionic currents in the sensory neurons, shifting downward the affinity of ASIC3 for protons, through a not yet clear combined activity on these channels [255]. In rats, subcutaneous administration of APETx2 exerted a potent analgesic effect on inflammation-induced hyperalgesia by peripherally blocking ASIC3 on cutaneous nociceptors [241]. Several other studies resorting to rodent models have demonstrated the efficacy of APETx2 in reducing postoperative, osteoarthritic, muscular, and inflammatory pain [241,256,257,258,259]. In rats, intraperitoneal injection of APETx2 before experimentally inducing acute gastric mucosal lesions reduced gastric acidity, mucosal injury, and ASIC3 expression in thoracic DRG projecting to the stomach, suggesting a role for ASIC3 in the development of gastritis symptoms [63]. More recently, APETx2 displayed a bell-shaped dose–response curve in reducing gut visceral pain evaluated by acetic-acid-induced abdominal contractile responses in mice [64]. In this study, the toxin exerted an analgesic effect only at lower doses, while its efficacy diminished at higher doses, possibly owing to the nonspecific action of the compound. An important drawback concerning the analgesic efficacy of APETx2 is represented by its off-target effects on Na_v_ 1.8, Na_v_ 1.6, Na_v_ 1.2, and cardiac hERG channels, which may influence its therapeutic potential [260,261]. Another important issue is represented by the ability of the toxin to partially modulate ASIC3 activation-mediated currents. The ability of APETx2 to reduce only a transient, but not a sustained, component of acid-induced ASIC3 current in whole cell patch clamp measurements in *Xenopus laevis* oocytes was considered a possible mechanism underlying its partial efficacy in modulating visceral pain. Indeed, another sea anemone toxin, Ugr9-1, which displayed a higher potency in reducing acidosis-associated pain conditions, was able to completely block ASIC3 currents in vitro [64,65]. The discovery of more selective and efficacious ASIC inhibitors represents an important challenge in order to target physiologically relevant and abnormally active ASICs involved in pathophysiological pain perception [262]. From this perspective, selective blockade of ASICs, in particular ASIC3, may provide interesting therapeutic tools to treat visceral hypersensitivity and pain associated with functional and inflammatory chronic gut diseases, such as IBS and IBD [239].

## 5. Conclusions

Visceral pain is a complex health problem, with increasing prevalence worldwide. Despite the growing body of literature, the pathogenic mechanisms underlying visceral pain are less well understood than in somatic pain. Neuronal nociceptive pathways are involved both in the peripheral and CNS, and a vast number of ion channels, such as Na_v_, Ca_v_, ASICs, and TRPs, and neurotransmitter pathways participate in visceral pain modulation. The possibility of targeting these molecular players along pain pathways may represent a useful approach to discover new analgesic drugs for visceral pain treatment. In this regard, a useful strategy would be represented by modulation of primary afferent neurons, which are the direct link between the gut and the CNS. Indeed, peripherally acting drugs may display a higher safety profile, with reduced blood–brain barrier permeability and passage into the CNS. This can be achieved by targeting proteins that are expressed selectively in peripherally located nociceptive neurons, or by synthesizing drugs with a reduced disposition within the CNS [8]. Marine toxins, by interacting with high potency and selectivity with diverse visceral pain molecular targets, including, among others, TRPV1, TRPA1, ASICs, VGSC and VGCC channels, and GABA_B_ receptors, display a potential antinociceptive profile. Indeed, numerous natural compounds, including marine toxins, are under preclinical and clinical investigations to test their therapeutic potential as analgesics, opening a new promising approach for pain therapy, including visceral pain treatment [263]. Some drawbacks should, however, be taken into consideration when considering marine toxins as possible pharmacological tools. For instance, the limited efficacy when targeting multiple molecular pathways involved in pain perception (i.e., ion channels or receptors), may address the use of toxins, or toxin-derived compounds, as adjuvant analgesics, more than single therapeutic agents [1,41]. Adjuvant analgesics are, however, of therapeutic importance since they may allow reduction of the dose of conventional analgesic, such as opioids, thus minimizing the possible adverse effects of these latter agents. Another major flaw hampering the clinical use of toxin peptides is their low oral bioavailability, which directs attention towards parenteral administration routes, with low patient compliance, high production cost, and low storage stability [264]. The most important strategies developed to overcome this latter issue comprise chemical modifications of the peptide structure or encapsulation of peptides into drug delivery systems to protect them from enzymatic degradation and to control their release [264,265]. Improvement of toxin peptide bioavailability would reduce drug doses and possible adverse side effects. Indeed, adverse effects and toxicity raise an important safety issue in relation to marine-toxin-based analgesic therapies [105,164,165]. Despite these drawbacks, identification of marine toxins with elevated selectivity for molecular pain targets may help improve our knowledge of the physiological and pathological mechanisms underlying pain perception, and may help design novel therapeutic agents, thus helping to ameliorate management of visceral pain associated with gut disorders.

## Figures and Tables

**Table 1:** Effects of marine toxins and related compounds in preclinical models of gut visceral pain.

Compound/Target	Dose/Concentration (Administration route)	Model	Parameter Evaluated	Effect	REF
TTX/Na_v_	1, 3, 6 g/kg (s.c.)	Intracolonic instillation of capsaicin in WT (C57BL/6 background) and Na_v_ 1.7 conditional KO	-Pain-related behaviors (licking of the abdomen, stretching of abdomen, abdominal retractions); -Referred mechanical hyperalgesia (response to abdominal stimulation with von Frey electrodes)	-Similar dose-dependent reduction in WT and KO -Similar dose-dependent reduction in WT and KO	[55]
	3, 6 g/kg (s.c.)	Intracolonic instillation of mustard oil in WT and Na_v_ 1.7 conditional KO mice	Pain-related behaviors (licking of the abdomen, stretching of abdomen, abdominal retractions)	Dose-dependent reduction, similar in WT and KO	[55]
	0.3, 1, 3, 6 g/kg (s.c.)	Intraperitoneal acetic-acid induced writhing test in Swiss Webster mice	Number of complete abdominal contractions accompanied with stretching of hind limbs	Dose-dependent reduction	[56]
ω-conotoxin MVIIA/Ca_v_ 2.2	1, 10, 30, 100 pmol/site (i.t.)	Intraperitoneal acetic-acid induced writhing test in Swiss mice	Number of complete abdominal contractions accompanied with stretching of hind limbs	Dose-dependent reduction	[57]
	1, 10, 30, 100 pmol/site (i.t.)	Intracolonic instillation of capsaicin in Swiss mice	Pain-related behaviors (licking of the abdomen, stretching of abdomen, abdominal retractions).	Dose-dependent reduction	[57]
	30 pmol/site (i.t.)	Intraperitoneal acetic-acid induced writhing test in Swiss mice	Measurement of glutamate levels in the CSF	Reduction of nociceptive stimulus-induced increase of glutamate levels in the CSF	[57]
	30 pmol/site (i.t.)	Intracolonic instillation of capsaicin in Swiss mice	Measurement of glutamate levels in the CSF	Reduction of nociceptive stimulus-induced increase of glutamate levels in the CSF	[57]
Vc1.1/GABA_B_	1 M (in vitro)	Human thoracolumbar DRG	Whole-cell patch clamp recordings	Inhibition of a selective population of DRG neurons	[58]
	1, 10, 100, 1000 nM (in vitro)	CVH mouse model induced by intrarectal TNBS administration	Ex vivo single fiber recordings of primary afferents splanchnic colonic and pelvic colorectum afferents	Concentration-dependent inhibition of mechanosensitivity (the effect was higher in CVH animals)	[58]
	1, 10, 100, 1000 nM (in vitro)	CVH mouse model induced by intrarectal TNBS administration	Ex vivo single fiber recordings of splanchnic colonic primary afferents.	Concentration-dependent inhibition of mechanosensitivity (the effect was higher in CVH animals)	[59]
	10 nM (in vitro)	CVH mouse model induced by intrarectal TNBS administration	Whole-cell patch clamp recordings on colonic extrinsic primary afferents	Significant inhibition of the excitability of colonic control DRG which was higher in CVH animals	[59]
	1 M (intrarectal enema)	CVH mouse model induced by intrarectal TNBS administration followed by noxious distension of the colorectum	VMR to colorectal distension by electromyography assessment	Significant reduction of VMR in CVH mice to noxious distension pressures	[60]
[Ser3] Vc1.1(1-8)/GABA_B_	100 pM, 30 nM, 1 M (in vitro)	Mouse DRG	Whole-cell patch clamp recordings	Inhibition of VGCC	[61]
	1, 10, 100 and 1000 nM (ex vivo, applied on the colonic surface)	CVH mouse model induced by intrarectal TNBS administration	In vitro single-unit extracellular recordings of action potential discharge from splanchnic colonic afferents.	Concentration-dependent inhibition of mechanosensitivity of splanchnic colonic primary afferents	[61]
	1 M (intrarectal enema)	Noxious distension of the mouse colorectum	VMR to colorectal distension by electromyography assessment	Significant reduction of VMR to colorectal distension vs vehicle treated animals	[61]
APCH1/TRPV1	0.5 mg/kg (i.v.)	Intraperitoneal acetic-acid induced writhing test in CD1 mice	Number of complete abdominal contractions accompanied with stretching of hind limbs	Reduction	[62]
APCH3/TRPV1	0.1, 0.5 mg/kg (i.v.)	Intraperitoneal acetic-acid induced writhing test in CD1 mice	Number of complete abdominal contractions accompanied with stretching of hind limbs	Reduction	[62]
APETx2/ASIC3	25 g/kg	Acute gastric mucosal damage induced by WIRS in Wistar rats	-intragastric pH -gastric histopathological changes -UI -ASIC3 expression in thoracic DRG neurons projecting to the stomach	Significant reduction of WIRS-induced: -gastric mucosal injury, UI score, gastric acidity -ASIC3 expression in DRG	[63]
	0.002, 0.02, 0.2, 1 mg/kg (i.m.)	Intraperitoneal acetic-acid induced writhing test in CD1 mice	Number of complete abdominal contractions accompanied with stretching of hind limbs	Bell-shaped reduction of abdominal contractile responses	[64]
Ugr9-1/ASIC3	−0.002, 0.02, 0.2, 1 mg/kg (i.m.); −0.5, 0.1, 0.01 mg/kg (i.v.)	Intraperitoneal acetic-acid induced writhing test in CD1 mice	Number of complete abdominal contractions accompanied with stretching of hind limbs	Dose-dependent reduction of abdominal contractile responses	[64,65]

Abbreviations: cerebrospinal fluid (CSF); chronic visceral hypersensitivity (CVH); trinitrobenzene sulphonic acid (TNBS); water immersion restraint stress (WIRS); visceromotor response (VMR).
